# Spatial distribution of tumor-associated macrophages in an orthotopic prostate cancer mouse model

**DOI:** 10.3389/pore.2024.1611586

**Published:** 2024-04-16

**Authors:** Korie A. Grayson, Joshua D. Greenlee, Lauren E. Himmel, Lauren A. Hapach, Cynthia A. Reinhart-King, Michael R. King

**Affiliations:** ^1^ Department of Biomedical Engineering, Vanderbilt University, Nashville, TN, United States; ^2^ Meinig School of Biomedical Engineering, Cornell University, Ithaca, NY, United States; ^3^ Department of Pathology, Microbiology and Immunology, Translational Pathology Shared Resource, Vanderbilt University Medical Center, Nashville, TN, United States

**Keywords:** tumor-associated macrophages, TRAIL, liposomes, prostate cancer, pathologic analysis

## Abstract

Mounting evidence suggests that the immune landscape within prostate tumors influences progression, metastasis, treatment response, and patient outcomes. In this study, we investigated the spatial density of innate immune cell populations within NOD.SCID orthotopic prostate cancer xenografts following microinjection of human DU145 prostate cancer cells. Our laboratory has previously developed nanoscale liposomes that attach to leukocytes via conjugated E-selectin (ES) and kill cancer cells via TNF-related apoptosis inducing ligand (TRAIL). Immunohistochemistry (IHC) staining was performed on tumor samples to identify and quantify leukocyte infiltration for different periods of tumor growth and E-selectin/TRAIL (EST) liposome treatments. We examined the spatial-temporal dynamics of three different immune cell types infiltrating tumors using QuPath image analysis software. IHC staining revealed that F4/80+ tumor-associated macrophages (TAMs) were the most abundant immune cells in all groups, irrespective of time or treatment. The density of TAMs decreased over the course of tumor growth and decreased in response to EST liposome treatments. Intratumoral versus marginal analysis showed a greater presence of TAMs in the marginal regions at 3 weeks of tumor growth which became more evenly distributed over time and in tumors treated with EST liposomes. TUNEL staining indicated that EST liposomes significantly increased cell apoptosis in treated tumors. Additionally, confocal microscopy identified liposome-coated TAMs in both the core and periphery of tumors, highlighting the ability of liposomes to infiltrate tumors by “piggybacking” on macrophages. The results of this study indicate that TAMs represent the majority of innate immune cells within NOD.SCID orthotopic prostate tumors, and spatial density varies widely as a function of tumor size, duration of tumor growth, and treatment of EST liposomes.

## Introduction

Prostate cancer (PCa) is the most common malignancy in men and the second leading cause of cancer-related death in American men [1]. Surgery, radiation, chemotherapy, and androgen-deprivation therapy (ADT) are the standard treatments administered at local or regional stages of disease [2]. Once the cancer has reached an advanced or metastatic stage that no longer responds to ADT, a stage known as castration-resistant prostate cancer (CRPC), treatment options become limited [3]. Due to the lack of curative therapies for advanced and metastatic disease, new anticancer therapeutics are needed to treat life-threatening metastases without the side effects of ADT, chemotherapy, and surgery.

It is widely accepted that inflammation is a hallmark of cancer [4, 5]. There is epidemiological and pathological evidence linking chronic inflammation with the etiology of PCa and the course of tumor progression [6–8]. Studies have demonstrated the importance of immune-tumor interactions along with the role of immune cell infiltration in tumor recurrence and overall survival in patients [9, 10]. Leukocytes are key components of tumorigenesis and are known to infiltrate tumors and drive cancer development via multiple signaling pathways. Many studies have connected prostatitis and PCa due to the frequent observation of inflammatory infiltrates in PCa lesions, although the source of intraprostatic inflammation remains unclear [11, 12]. The most well studied infiltrating immune cells in PCa to date have been T cells (CD4^+^, CD8^+^, Th17), B cells, macrophages, mast cells, immunosuppressive cells (Tregs, MDSCs), neutrophils, and natural killer cells [13, 14]. Studies on biopsy tissues from PCa patients across different stages have correlated pro-tumorigenic or anti-tumorigenic inflammatory responses with overall survival. One study showed that very low or very high CD3^+^ T cell infiltration correlated with PSA recurrence-free survival in prostate cancer patients [15]. In other studies, patients treated with androgen deprivation therapy had higher densities of CD3^+^ and CD8^+^ lymphocytes with no correlation to biochemical occurrence [16]. The highlighted role of T lymphocytes associated with PCa and its progression has led to two FDA-approved immunotherapies, Sipuleucel-T and pembrolizumab, along with many others in preclinical studies [17, 18]. Most immunotherapies are T cell focused and do not take advantage of innate immune cells in treating prostate cancer. However, recent preclinical studies and early clinical trials suggest that myeloid cells such as macrophages are an emerging target of novel anticancer therapies [19].

Macrophages are one of the most abundant immune cells in PCa tumors [20, 21]. When infiltrating tumors, macrophages are termed tumor-associating macrophages (TAMs). TAMs are traditionally classified into two types based on different activation pathways: classically activated (M1) and alternatively activated (M2) macrophages which possess anti-tumor or pro-tumor functionality, respectively [22]. Accumulating evidence suggests an oversimplification of binary M1/M2 labeling for macrophages, and that they more accurately exist on a spectrum between pro-inflammatory (M1) and wound healing (M2) [23]. TAM ontology and regional associations within tumors are areas currently being explored to explain their role in tumor immunity and progression. Additionally, the phenotypic heterogeneity of TAMs has spurred research into therapeutic agents to control macrophage polarization for therapeutic benefit [24]. For example, CSF1R blockade via small molecule agonists or monoclonal antibodies have been shown to reprogram TAMs toward an antitumor phenotype in preclinical studies [25, 26].

Tumor necrosis factor-related apoptosis inducing ligand (TRAIL) is a type II transmembrane protein that can be cleaved from the cell surface to produce its soluble form. TRAIL selectively induces apoptosis in cancer cells via binding to transmembrane death receptors (DR4, DR5) [27]. Death receptors cluster and then recruit Fas-associated death domain (FADD), which in turn activates caspase-8. Caspase-8 activates two pathways, one through the mitochondria causing cytosolic release of cytochrome c, and a more direct route which signals directly to the executioner caspase-3 [27]. TRAIL is expressed in a variety of immune cells, such as natural killer cells, and has shown therapeutic utility *in vitro* and *in vivo* [28, 29]. However, TRAIL’s clinical implementation has been confounded by a lack of bioavailability, and mechanisms of tumor cell resistance [30, 31].

Our lab has previously designed and tested a liposomal formulation designed to target circulating tumor cells (CTCs) via conjugation of E-selectin, an adhesion molecule expressed by activated endothelium and involved in metastasis and inflammation, and TRAIL [32]. These E-selectin/TRAIL (EST) liposomes have been shown to kill a variety cancer cells in the circulation by functionalizing leukocytes with TRAIL via E-selectin receptor adhesion [32–34]. These EST liposomes have also been tested in orthotopic xenograft tumor models of the prostate and breast [35, 36]. Orthotopic injection of cancer cells into the prostate has become a commonly used method to understand tumor progression and treatment efficacy over the past decade [37, 38]. These models are advantageous as they have been shown to metastasize to clinically relevant foci and better recapitulate the tumor microenvironment compared to subcutaneous tumors [39]. TRAIL-coated leukocytes were found to block widespread metastasis and increase survival in NOD.SCID orthotopic models of prostate and breast cancer [35, 36]. While these studies demonstrated that EST liposomes bind to leukocytes in the circulation, it remains to be determined how these liposomes affect immune cell infiltration into orthotopic tumors.

The aim of the present study was to investigate both the type and distribution of different innate immune cell subsets within NOD.SCID orthotopic PCa tumors in response to EST liposome treatment. An additional aim was to identify how the spatial distribution of immune cells, specifically macrophages, changes between early and late-stage tumors. Some prior studies have examined the location and distribution of specific leukocytes in human and mouse prostate tumors [40–42]. However, this is the first study to examine the spatial distribution of immune cells within a NOD.SCID orthotopic PCa mouse model and investigate innate immune cell infiltration in response to liposome treatments.

## Materials and methods

### Transduction of DU145 human prostate cancer cells

DU145 human prostate cancer cells (ATCC #HTB-81) were obtained from American Type Culture Collection (Manassas, VA, United States). The cells were maintained in Eagle’s Minimum Essential Medium (EMEM) (Corning, Corning, NY, United States). Media was supplemented with 10% fetal bovine serum and 1% penicillin (Gibco, ThermoFisher, Waltham, MA, United States). DU145 cells were transfected with a pCMV lentivirus containing mCherry and D-luciferase (LP466-025) markers along with a puromycin gene (GeneCopoeia, Rockville, MD, United States). Infected cells were selected with puromycin (P8833, Sigma Aldrich, St. Louis, MO, United States) for puromycin resistance and were cultured according to ATCC guidelines. To confirm the stability of transduction, cells were imaged using an Olympus IX81 epifluorescence microscope to detect mCherry expression/fluorescence. Bioluminescence imaging (BLI) using a Xenogen IVIS 200 Imaging System was used to confirm luciferase expression.

### Prostate orthotopic implantation

All experimental procedures were approved by the Vanderbilt University Institutional Animal Care and Use Committee and were conducted in an AAALAC-accredited facility in accordance with the Guide for the Care and Use of Laboratory Animals and the Public Health Service Policy on Humane Care and Use of Laboratory Animals. Male NOD.CB17-Prkdc^scid^/J mice 6–8 weeks old (#001303, Jackson Laboratory, Bar Harbor, ME, United States) were placed under anesthesia using 5% isoflurane and then reduced to 2% after animals were completely anesthetized. Mice were dehaired using a depilatory cream and a sterile cotton swab. The area was cleaned with 70% ethanol. After removing the hair, the area was cleaned using iodine and 70% ethanol swabs 3 times each. A low midline abdominal incision about 1–3 mm long was made through the skin and muscle layer using a sterile scalpel. The bladder was exteriorized from the body and the ventral lobes of the prostate were located. Using a 30G needle, 1 million D-luciferase:mCherry labeled DU145 cells suspended in 30 μL PBS were injected into the ventral prostate. The muscle layer was closed with 4–0 absorbable sutures. The skin layer was closed with 5–0 non-absorbable sutures. Animals were monitored every 24 h and given analgesic medication for 2 days post-surgery.

### Preparation and injection of EST liposomes

Multilamellar liposomes, composed of egg L-α-lysophosphatidylcholine (Egg PC, #840051C), egg sphingomyelin (Egg SM, #86001C), ovine wool cholesterol (Chol, #700000P), and 1,2-dioleoyl-sn-glyc- ero-3- [(N-(5-amino-1-carboxypentyl) iminodiacetic acid) succinyl] (nickel salt) (DOGS NTA-Ni, #790404C) at weight ratios 50%:30%:10%:10% (Egg PC/Egg SM/Chol/DOGS NTA-Ni), were prepared using a thin lipid film method (Avanti Polar Lipids, Alabaster, AL). DOGS-NTA-Ni is a lipid conjugated to nickel-nitrilotriacetic acid (Ni-NTA) that allows for attachment to histidine-tagged proteins. Briefly, stock solutions of all of the lipids were prepared by dissolving powdered lipids in chloroform to produce a final concentration of 5 mg/mL Egg PC, 20 mg/mL Egg SM, 5 mg/mL Chol, and 20 mg/mL DOGS-NTA-Ni in glass containers and stored at −20°C. Appropriate volumes of the lipids were taken from the stock solution to prepare the lipids in a glass tube. TopFluor^®^ Cholesterol was also added to the lipid mix to fluorescently label liposomes. Lipids were gently dried under vacuum for 12 h to remove chloroform. The lipid film was hydrated with a liposome buffer composed of 150 mM NaCl, 10 mM Hepes, and 1 mM MgCl_2_ dissolved in nuclease-free water to create multilamellar liposomes. The resulting multilamellar liposomes were resized by repeated thawing and freezing, and then subjected to 10 extrusion cycles at 55°C through two different pore size (200 and 100 nm, #WHA800281 and #WHA800309) polycarbonate membranes (Sigma Aldrich, St. Louis, MO, United States) to produce unilamellar nanoscale liposomes. Liposomes were conjugated with E-selectin and TRAIL for 30 min at 37°C and then stored overnight at 4°C. Soluble histidine-tagged TNF-related apoptosis-inducing ligand (TRAIL) (BML-SE721-0100) was purchased from Enzo Life Sciences (Farmingdale, NY, United States). Histidine-tagged recombinant human E-selectin (724-ES) was purchased from R&D Systems (Minneapolis, MN, United States). Mice in the 3 weeks (treated) group received one tail-vein injection of EST liposomes (0.002 mg/kg) occurring at three-weeks post tumor implantation. Mice in the 6 weeks (treated) group received liposome injections once every 3 days in alternating veins occurring at three-weeks post tumor implantation until week 6 (6 doses total). Mice were humanely sacrificed via CO_2_ asphyxiation 2 days following the last injection of liposomes.

### Bioluminescence imaging

Post tumor implantation, animals were monitored weekly for bioluminescent activity. Luciferin was administered at 150 mg/kg via intraperitoneal injection using a 30G insulin syringe needle. Animals were placed under anesthesia using 2% isoflurane and imaged 5 min post injection for maximum bioluminescence signal. Images were taken at 1 s exposure time using a Xenogen IVIS 200 Imaging System. For the quantitative measurements of average radiance, the area of the orthotopic prostate xenograft BLI signal was constant throughout all time points for each animal.

### Histology

Pathology endpoints were carried out in the Vanderbilt Translational Pathology Shared Resource (TPSR). Mice were euthanized by CO_2_ asphyxiation, in accordance with AVMA Guidelines for the Euthanasia of Animals. Complete necropsy was performed, and body weights and organ weights were recorded. Prostatic xenograft length and width were measured with calipers, and volume was calculated using the equation V = (L × W^2^)/2. Tissues were fixed in 10% neutral buffered formalin for 72 h. Fixed tissues were routinely processed using a standard 8-h processing cycle of graded alcohols, xylenes, and paraffin wax, embedded and sectioned at 4–5 microns, floated on a water bath, and mounted on adhesive glass slides. Hematoxylin and eosin (H&E) staining was performed on a Gemini AS Automated Slide Stainer (ThermoFisher Scientific, Waltham, MA).

### Immunohistochemistry

Immunohistochemical (IHC) staining for innate immune cell infiltrates was performed using macrophage (F4/80), monocyte and granulocyte (CD11b), neutrophil and monocyte (MPO), eosinophil (MBP), and neutrophil (Neutro) antibodies on a Leica Bond-Max automated stainer (Leica Biosystems Inc., Buffalo Grove, IL, United States). All five antibodies used for immunophenotyping in this study are validated, on-demand stains maintained in the Translational Pathology Shared Resource (TPSR) by regular quality assurance. All steps besides dehydration, clearing, and cover slipping were performed on the Bond-Max. Immunolabeling was conducted using antibodies listed in Supplementary Table S1. The Bond Polymer Refine Detection system was used for visualization. Where a rat primary antibody was used, a rabbit anti-rat secondary antibody was substituted in the Bond Polymer Refine Detection kit, and the rest was used as specified by the manufacturer. Slides were then dehydrated, cleared, and cover slipped. All histopathologic interpretation was conducted by a board-certified veterinary pathologist under masked conditions. Slide scanning was performed on the Pannoramic 250 Flash III digital scanner (3DHISTECH Ltd., Budapest, Hungary). Quantification of immunolabeled cells in the tumors was performed using manual region of interest (ROI) delineation of the tumor and a single-threshold positive cell detection feature. Orthotopic prostate xenografts were manually outlined and then a margins script was applied to distinguish between ROIs. A smoothing feature was applied to create a heatmap visualization of leukocyte density in terms of these ROIs for each leukocyte marker. The distribution of immune cell infiltrates in the periphery vs. the inner portions of the tumors was assessed. The periphery was defined as the outer ¼ of the radius of a mass; the inner region was denoted as the remaining ¾ of the radius of a mass. Data were collected per tumor section and then group-wide means of percentage of positively stained cells were calculated.

To identify apoptotic cells in tumor sections, terminal deoxynucleotidyl transferase dUTP nick end labeling (TUNEL) staining was performed. Slides were placed on a Leica Bond RX IHC stainer (Leica Biosystems Inc.). All steps besides dehydration, clearing, and cover slipping were performed on the Bond RX. Slides were deparaffinized. Antigen retrieval was performed on the Bond RX using Triton X-100 (Cat#T9284, Sigma-Aldrich, St. Louis, MO) for 5 min. Slides were incubated with Equilibration Buffer (#G7130, Promega, Madison, WI, United States) for 5 min, followed with the TdT reaction mix (#G7130, Promega) for 10 min and SSC-x20 (#G7130, Promega) for 10 min. The slides were incubated with streptavidin-HRP (#RE7104, Novocastra, Newcastle Upon Tyne, United Kingdom) for 5 min, and the Bond polymer refine detection system (#DS9800, Leica Biosystems Inc.) was used for visualization. Slides were then dehydrated, cleared, and cover slipped. Slide scanning was performed on a Pannoramic 250 Flash III digital scanner. Quantification of immunolabeled cells in the tumors was performed by a board-certified veterinary anatomic pathologist in QuPath (Supplementary Figures S5, S6), an open source software for digital pathology image analysis, using a script for manual region of interest delineation of the tumor and a single-threshold positive cell detection feature [43, 44]. Data were collected per tumor section and then group-wide means of the percentage of TUNEL-positive cells were calculated.

### Confocal microscopy

For liposome and leukocyte infiltration imaging, orthotopic prostate xenografts were removed from all animals after sacrifice. Xenografts were embedded in OCT compound on dry ice and sectioned at 4–5 microns, and then mounted on adhesive glass slides. Samples were fixed with 4% paraformaldehyde (PFA) in PBS for 10 min at room temperature (RT). Slides were subsequently washed with wash buffer (PBS + 0.02% Tween 20) three times for 5 min each at RT. Samples were then permeabilized using PBS + 1% Triton for 5 min at RT. Slides were washed 3X. Slides were blocked with blocking buffer (PBS + 10% FBS + 5% donkey serum 5% goat serum) for 2 h at RT. Rat mAb F4/80 primary antibody (#NB600-404, Novus Biologicals, Littleton, CO, United States) was diluted 1:500 into blocking solution. Slides were incubated with primary antibody solution overnight at 4°C in a humidified chamber. After primary antibody staining, samples were washed 3X on the following day. Anti-rat Alexa Fluor^®^ 647 Conjugate secondary antibody (#4418S, Cell Signaling Technology, Danvers, MA, United States) was diluted 1:500 into blocking solution and all samples were stained with secondary antibody for 2 h at RT in the dark. Slides were washed 3X. Samples were then stained with DAPI for 30 min followed by washing 3X at RT in the absence of light. Mounting medium (#H-1000, Vector Labs, Burlingame, CA, United States) was added followed by a coverslip, then the edges were sealed with nail polish. Confocal z-stacks of immunofluorescence-stained histology samples were obtained using a Zeiss LSM 800 inverted confocal microscope equipped with a 40X/1.1 N.A. long working distance water-immersion objective and a 100X/1.46 N.A oil immersion objective using 405, 488, 561, and 640 laser lines and operated by Zen 2.3 software. Maximum z-projections were used to assess liposome tumor infiltration and macrophage localization. Photomicrographs were processed in ImageJ.

### Statistical analysis

GraphPad Prism 8 software (San Diego, CA, United States) was used to plot and analyze data sets. Two-tailed unpaired *t*-test was used for comparisons between two groups, with *p* < 0.05 considered significant. ANOVA with Tukey post-test was used for comparing multiple groups with *p* < 0.05 considered significant. Shapiro-Wilk test was used to test normality. Data are presented as mean ± SD with at least three independent replicates used for each experiment. A minimum sample size of n = 3 mice per group was calculated using mean and standard deviation data from our previous TRAIL liposome treatment study, assuming α = 0.05 and power = 80% [35].

## Results

### Total leukocytes in prostate tumors decrease over time and in response to TRAIL liposomes

**FIGURE 1:**
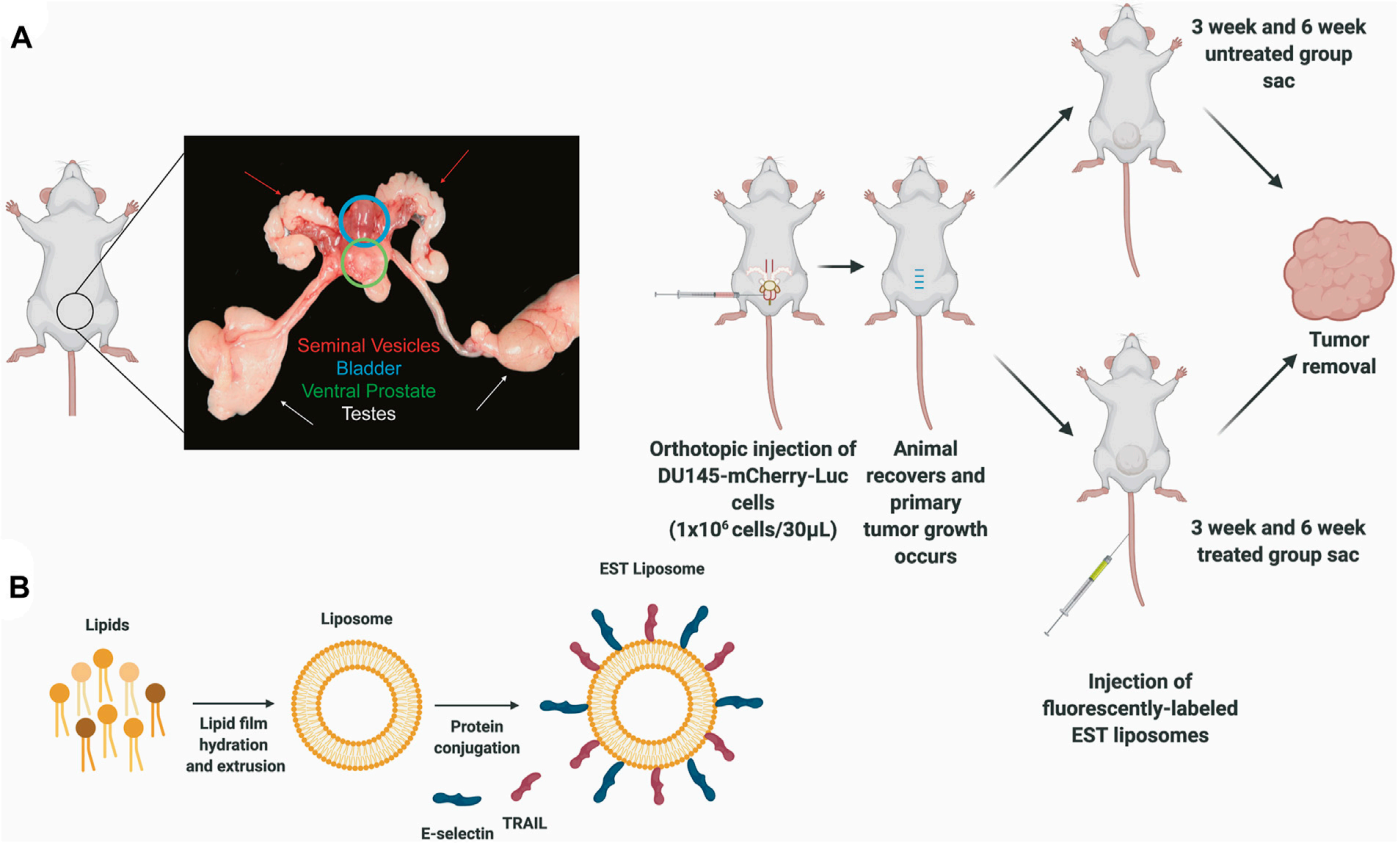
Protocols for inoculating orthotopic prostate xenografts and preparing E-selectin/TRAIL (EST) liposomes. **(A)** Schematic of orthotopic xenograft model for whole tumor analysis depicting an anatomical picture of the male mouse reproductive system to denote location of ventral prostate with tumor. **(B)** Preparation of E-selectin/TRAIL liposomes.

To identify and quantify the types of mouse leukocytes that infiltrate orthotopic prostate xenografts, dual labeled DU145-mCherry-Luc human prostate cells were orthotopically injected into the ventral prostate of NOD.SCID mice (Figure 1A). Mice were sorted by primary tumor size, as measured by BLI, at week two and size-matched into four groups: 3 weeks (Untreated) (n = 4), 3 weeks (Treated) (n = 3, 1 injection of EST liposomes), 6 weeks (Untreated) (n = 4), and 6 weeks (Treated) (n = 4, 6 injections of EST liposomes) (Figures 1A, B). Interestingly, EST liposome treatments had no significant effect on terminal tumor volume or tumor burden measured through bioluminescence imaging (Supplementary Figure S1). Terminal body weight, and relative organ weights of the liver, heart, spleen, and kidneys showed no significant difference among cohorts (Supplementary Figure S2). Metastasis was not observed in any group, and all subsequent analysis was completed using orthotopic xenograft tumors of the prostate.

**FIGURE 2:**
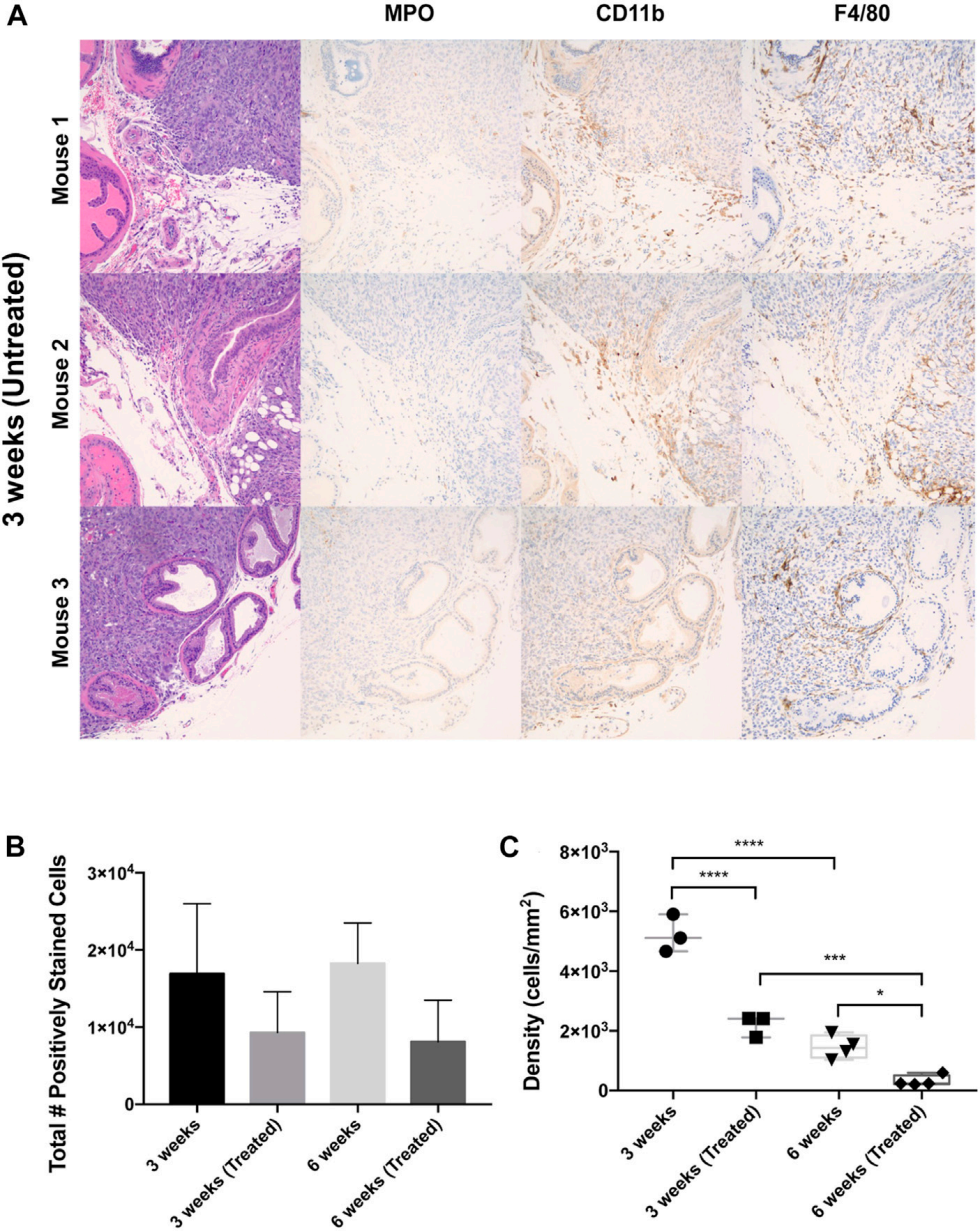
Leukocyte populations within orthotopic prostate tumors across liposome treatments and endpoints. **(A)** IHC staining of mouse ventral prostate tissue subjected to orthotopic tumor engraftment. MPO = myeloperoxidase/neutrophil granulocyte marker, CD11b = myeloid marker, F4/80 = macrophage marker. Images show the interface of the ventral prostatic xenograft with adjacent normal prostate and adipose tissue in three mice from the 3 weeks untreated group. All photomicrographs taken at ×200 magnification. **(B)** Total number of F4/80+, CD11b+, MPO + cells in whole tumor section for each treatment cohort. **(C)** Overall leukocyte density across treatment groups. The values represent the mean ± SD (n = 3 or 4). *p < 0.05, ***p < 0.001, ****p ≤ 0.0001.

Immunophenotyping of tumoral leukocytes was accomplished via immunohistochemical staining with F4/80, CD11b, and MPO markers (Figure 2A). F4/80 is a well-characterized and highly cited mouse macrophage marker expressed at high levels in various tissues [45]. CD11b is a common myeloid marker for monocytes and granulocytes [46], and myeloperoxidase (MPO) is a marker for neutrophil granulocytes [47]. Tumors were also stained for eosinophils (MBP) and an additional marker for neutrophils (Neutro) but only exhibited < 1% positively stained cells, so these markers were not studied further. To establish a baseline of leukocyte infiltration caused by surgery alone, a control group of mice was subjected to sham surgery involving an injection of PBS administered to the ventral prostate. These non-tumor bearing mice displayed infrequent prostatic inflammatory infiltrates with F4/80, CD11b, and MPO positive staining of less than 2% each (Supplementary Figure S3). Interestingly, the total number of leukocytes (total F4/80, CD11b, and MPO cells combined) detected in orthotopic prostate xenografts decreased in EST liposome-treated animals by 46% at the 3-week timepoint and by 55% at 6 weeks (Figure 2B). This observation suggests that EST liposomes have a measurable effect on the degree of leukocyte infiltration within these orthotopic prostate tumors. Additionally, total leukocyte density in xenografts showed not only a decrease in EST liposome-treated animals, but also decreased over time from 3-week to 6-week tumors (Figure 2C). In untreated tumors, leukocyte density decreased significantly from ∼5200 cells per mm^2^ at 3 weeks to ∼1,400 cells per mm^2^ after 6 weeks. A significant decrease in leukocyte density was also observed in EST liposome-treated tumors between 3-week and 6-week timepoints. Similar to treatment trends observed from total leukocyte counts, leukocyte density significantly decreased in response to liposome treatments. The discrepancies between total positive staining and density can be attributed to the difference in tumor sizes seen in each group (Supplementary Figure S1).

### Macrophages are the most abundant infiltrating immune cell type within orthotopic PCa tumors

**FIGURE 3:**
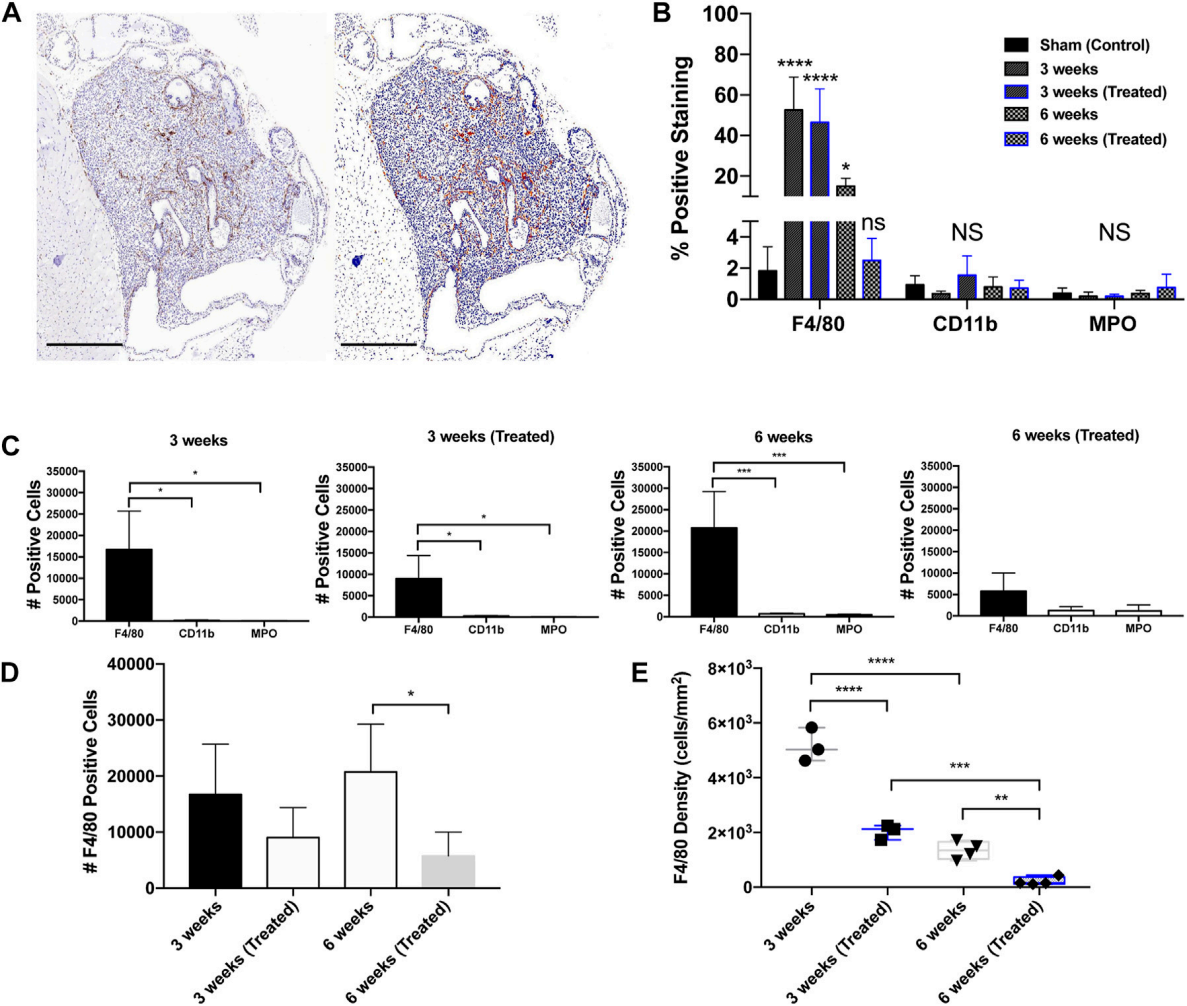
Immunohistochemical staining in orthotopic mouse prostate tumors. **(A)** Prostatic xenograft tumor from a 3-week untreated mouse (40X). The left panel shows a digital photomicrograph of F4/80 IHC. The right panel demonstrates a positive pixel count image analysis mock-up that enables quantitative analysis of DAB immunohistochemical staining. Scale bar = 500 μm. **(B)** Positive staining expression in all groups including control (n = 3). **(C)** Positive staining for leukocyte markers in different treatment groups: 3 weeks untreated (n = 3), 3 weeks treated (n = 3), 6 weeks untreated (n = 4), and 6 weeks treated (n = 4). **(D)** F4/80+ TAM positive cell counts across groups. **(E)** F4/80+ TAM density across groups. The values represent the mean ± SD (n = 3 or 4). *p < 0.05, **p < 0.01, ***p < 0.001, ****p ≤ 0.0001.

IHC analysis on whole tumor sections revealed that macrophages were the most abundant infiltrating immune cell type in all groups. The predominant immune infiltrates were mature macrophages, indicated by significant F4/80-positive staining (Figures 3A, B). When compared to the sham control (1.8%), tumors showed a significant increase in F4/80+ cells in the 3 weeks (Untreated) (52.6%), 3 weeks (Treated) (46.5%), and 6 weeks (Untreated) (15.0%) groups. Infiltration of CD11b+ and MPO+ cells was negligible in all treatment groups compared to the tumor-free control prostate tissue (Figures 3B, C). Based on these findings, all subsequent analysis was done solely for F4/80+ staining to measure differences across timepoints and EST liposome treatments. F4/80+ cell counts and density measurements followed similar trends to total leukocytes since macrophages were found to make up the majority of tumor immune cells. The number of F4/80+ cells decreased in EST liposome-treated animals by 46% at 3 weeks and 72% at 6 weeks (Figure 3D). Macrophage density decreased significantly with time and in tumors from animals administered EST liposomes: 3 weeks (∼5100 cells/mm^2^), 3 weeks (Treated) (∼2000 cells/mm^2^), 6 weeks (∼1,300 cells/mm^2^), 6 weeks (Treated) (∼210 cells/mm^2^) (Figure 3E). There was a significant decrease in the abundance of macrophages in the 6 weeks (Treated) group (receiving 6 liposome injections) compared to the 3 weeks (Treated) group (only one liposome injection), suggesting that sustained EST liposome injections decrease the number of TAMs within these prostate tumors.

### Spatial distribution of TAMs changes according to tumor progression and EST liposome treatments

**FIGURE 4:**
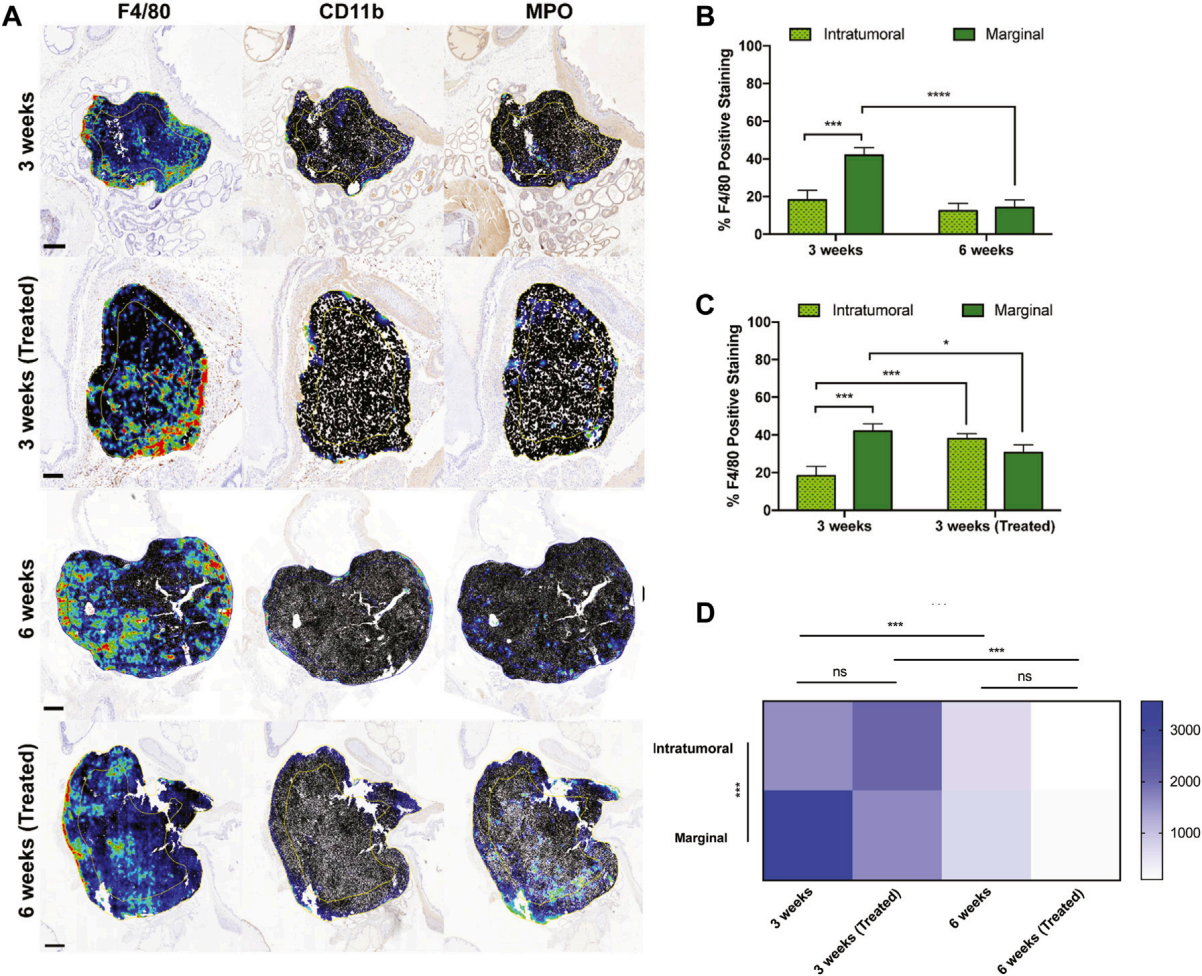
Intratumoral versus marginal distribution of F4/80+ cells in each treatment group. **(A)** Digital micrographs of F4/80, CD11b, and MPO IHC stain depicting two regions of interest to define the “intratumoral” versus “marginal” tumor microenvironments. Heatmaps were generated from positive cell detection image analysis of IHC stains for immune cell markers. Yellow/blue lines within each tumor recapitulate the perimetric shape and demarcates the “margin” area from the “center.” Scale bars = 500 μm, 1 mm, 2 mm, respectively. **(B,C)** Percent positively stained F4/80+ cells represented intratumorally and marginally at different time points and treatments. **(D)** TAM density among ROIs and treatment cohorts. *Y*-axis is cells/mm^2^. The values represent the mean ± SD (n = 3 or 4). NS = non-significant difference, *p < 0.05, ***p < 0.001, ****p ≤ 0.0001.

To interrogate the spatial distribution of TAMs in orthotopic prostate xenografts, two regions of interest (ROIs) were annotated to denote the marginal and intratumoral regions. F4/80 detection in each group displayed greater density compared to CD11b and MPO, which were both primarily located at the tumor margin (Figure 4A). In all four groups, macrophages were widely distributed throughout both tumor ROIs but were more densely concentrated near the tumor margin than intratumorally at 3 weeks of tumor growth (Figure 4B). After 6 weeks, there was a significant decrease in the number of marginal macrophages, but interestingly this did not coincide with an increase in intratumoral macrophages. When comparing the effects of treatment at the 3-week timepoint, the number of marginal macrophages decreased but intratumoral macrophages increased (Figure 4C).

The marginal and intratumoral spatial densities of macrophages in each treatment group were also explored. In early untreated tumors, TAM density was most concentrated at the tumor margins (Figure 4D). However, the ratio of marginal-to-intratumoral macrophages decreased in response to liposome treatment and time. TAM densities also differed in the untreated and treated groups at 3 and 6 weeks, demonstrating a significant decrease in macrophage quantity over time of tumor growth. These findings suggest that the spatial infiltration of macrophages is dependent on time, tumor growth, and EST liposome treatments. This is supported by macrophage density measurements in the 6 weeks treatment group, which showed the lowest density in both ROIs and compared to all other groups.

### EST liposomes functionalized to TAMs induce apoptosis in orthotopic PCa tumors

**FIGURE 5:**
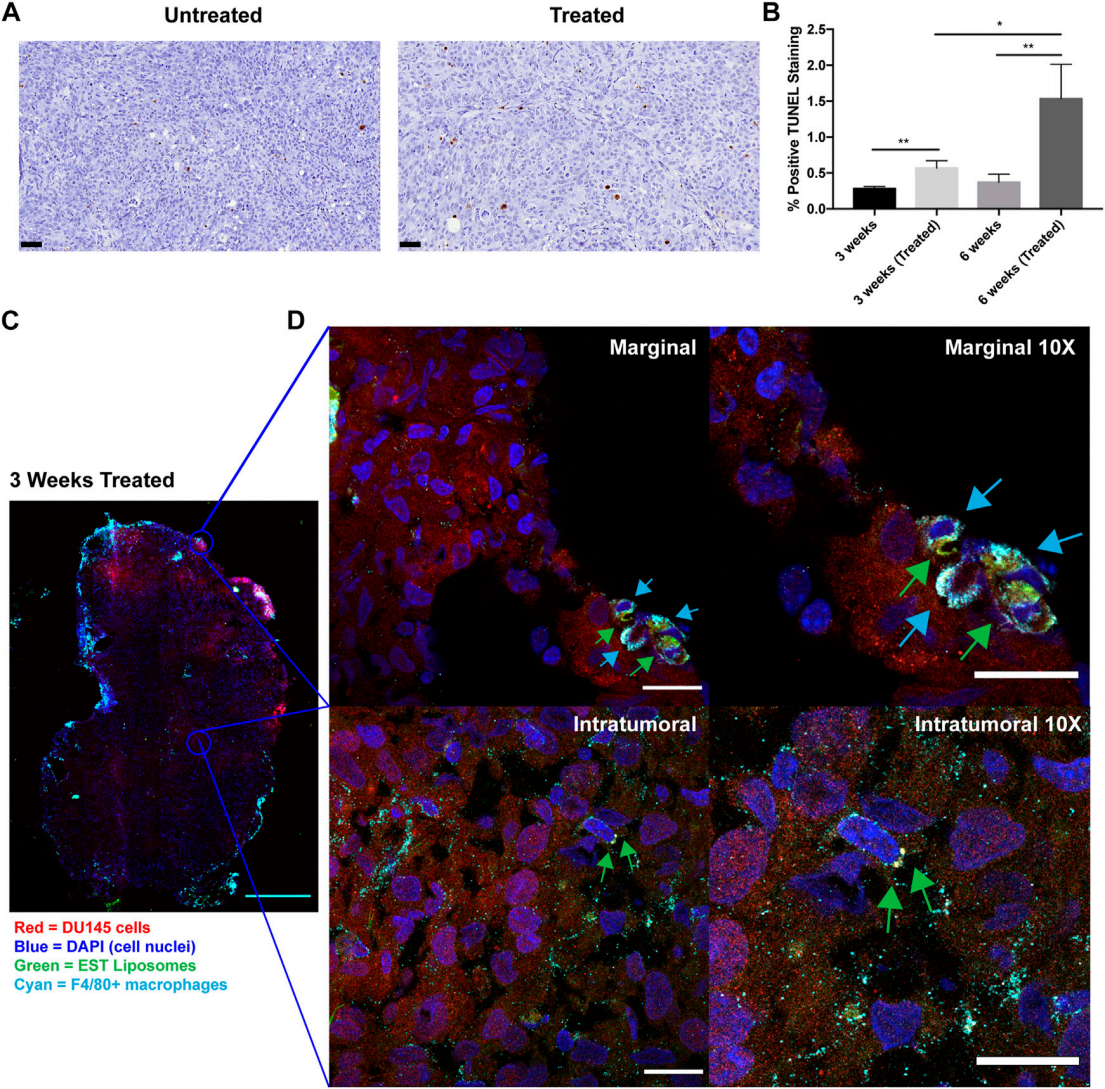
TRAIL-coated leukocytes bind to macrophages and increase apoptosis in treated tumors. **(A)** Representative annotated prostatic xenografts from 3 weeks (Untreated) and 3 weeks (Treated) animals with TUNEL IHC stain. Scale bar = 50 μm. **(B)** Percent positive TUNEL cells in each treatment group. **(C)** Confocal immunofluorescence analysis of intratumoral and marginal TRAIL-coated TAMs in the 3 weeks treated group. Cancer cells were labeled with mCherry (red), macrophages were labeled with F4/80 Alexa Fluor^®^ 647 Conjugate (cyan), cell nuclei were labeled with DAPI (blue), EST liposomes were labeled with TopFluor (green). Scale bar = 2 mm. **(D)** Representative images of TRAIL-coated TAMs (×1 and ×10 magnification) at the periphery and center of an orthotopic prostate xenograft. Blue arrows represent F4/80+ macrophages and green arrows represent EST liposomes. Scale bars = 200 μm and 20 μm, respectively. The values represent the mean ± SD (n = 3 or 4). *p < 0.05, **p < 0.005.

To determine whether this liposomal delivery method of TRAIL was inducing cellular apoptosis in orthotopic prostate xenografts, TUNEL staining was performed on tumor sections from each treatment group (Figure 5A). This assay detects DNA fragmentation that occurs in late-stage apoptosis. Mice treated with EST liposomes saw a significant increase in apoptotic cells compared to untreated mice (Figure 5B). Notably, just one injection of liposomes at 3 weeks increased apoptotic cells by over two-fold. Mice treated with six injections in the 6 weeks treated group saw an even greater effect with an over four-fold increase in apoptotic cells compared to untreated tumors.

To evaluate whether EST liposomes adhere to TAM populations in solid tumors, we used confocal microscopy to examine TRAIL-coated TAM presence in xenografts. The periphery and center of 3 weeks treated tumors were imaged to visualize EST liposome and F4/80+ cell interactions (Figure 5C). Tumors from the 6-week timepoint were too large to conduct whole tumor fluorescence confocal microscopy in this manner. Negative control confocal micrographs for 3 weeks untreated tumors with F4/80+ TAMs were also taken to confirm positive identification of liposomes (Supplementary Figure S4). In treated tumors, the majority of TAMs were located at the tumor margins, and EST liposomes were positively identified on the surface of these macrophages (Figure 5D). Intratumorally, fewer F4/80+ cells were present, however liposome-coated TAMs and free EST liposomes were also identified within the core of the tumor (Figure 5D). Collectively, these findings demonstrate that EST liposomes are capable of attaching to TAMs, infiltrating orthotopic prostate tumors, and increasing apoptosis of tumor cells.

## Discussion

Here, we report the distribution of infiltrating leukocytes in an orthotopic prostate cancer model while demonstrating a nanoscale immunotherapy approach to create TRAIL-coated leukocytes that can induce apoptosis in solid tumors. Similar to our 2016 study, we used an orthotopic prostate cancer model to study how immune cells infiltrate orthotopic prostate xenografts with EST liposomes as surface-tethered cargo [35]. We have previously demonstrated that E-selectin/TRAIL conjugated liposomes bind to leukocytes in the blood circulation and bombard circulating tumor cells (CTCs) to induce programmed cell death [32–34]. We hypothesized that these same TRAIL-coated leukocytes not only kill CTCs in the circulation, but can also hitchhike on the surface of immune cells to increase apoptosis in solid tumors. Encouragingly, we have demonstrated that this approach is effective in a variety of animal models with no off target toxicity to the liver or other organs [32, 35, 36, 48].

We first identified different populations of immune cells within orthotopic xenografts in an immunodeficient mouse model. Because NOD.SCID mice lack functional T cells, B cells, and natural killer cells, we focused on leukocytes in the innate immune arm, of which macrophages were found to be the most prevalent. Neutrophils were found in very low quantities within tumors despite accounting for approximately 50% of the white blood cell count in NOD.SCID mice [49]. However, neutrophils have the shortest half-life of all immune cells, 6–11 h, which may account for why their presence is minimal within these tumors [50–52]. Macrophages are a diverse population of cells, making characterization difficult using one broad marker. Macrophages can either be derived from monocytes originating from hematopoietic stem cells, or can be embryonic, characteristic of quiescent tissue-resident macrophages. Some studies have shown that F4/80^**high**^ macrophages are derived from the yolk sac, while F4/80^**low**^ macrophages derive from hematopoietic stem cells [53]. We used the F4/80 marker because it is a widely accepted mature macrophage marker; however, future studies should include a multiplicity of markers using flow cytometry and/or immunofluorescence to denote ontogeny of macrophage populations, as well as M1/M2-like characteristics across tumor microenvironments and treatments. In this study, it is possible that pro-tumor polarization of macrophages towards an M2 phenotype is occurring. An indicator could be the large increase in tumor volume from 3 weeks to 6 weeks. We also demonstrate throughout this study that macrophage infiltration decreases with time and increasing tumor size, which supports previous work that demonstrated the extent to which infiltration of TAMs was inversely associated with clinical stage [54]. Future studies should further investigate M1/M2 polarization within early and late-stage tumors to identify mechanisms that drive hot versus cold prostate tumors.

Tumors treated with E-selectin/TRAIL liposomes consistently saw decreased macrophage counts compared to untreated tumors. Treated tumors also demonstrated increased apoptosis as verified by TUNEL staining. This indicates that EST liposomes may be inducing TRAIL-mediated apoptosis in TAMs in addition to tumor cells. There is increasing evidence that macrophages, similar to tumor cells, express functional death receptors and are sensitive to TRAIL-induced apoptosis [55, 56]. Macrophages display particularly high death receptor expression and TRAIL sensitivity compared to other immune cells such as lymphocytes and neutrophils. The increase in TUNEL staining in treated mice may be explained by apoptotic macrophages present in these xenografts. This also correlates with the decrease in macrophage numbers seen in treated groups. Additionally, macrophage infiltration density decreased as a response to tumor progression and liposome treatment which might also be explained by TAMs displaying some sensitivity to TRAIL liposomes. Future studies should investigate mechanisms of TRAIL-mediated apoptosis in tumor-associated macrophages, especially between M1 and M2 phenotypes.

In contrast to our 2016 orthotopic prostate tumor study, TRAIL liposomes were unable to reduce primary tumor volume [35]. Recent data suggests DU145 cells are TRAIL resistant and show very minimal apoptosis in 2D and 3D cultures when treated with TRAIL alone [57, 58]. Tumor cells can also develop mechanisms to avoid TRAIL cytotoxicity [55]. Additionally, we hypothesize that minimal treatment effects were observed due to subtherapeutic liposome dosing as a consequence of changing TRAIL manufacturers over the years. In future studies, we anticipate a higher dosage of TRAIL with minimal injections will yield more significant results, as evidenced by our 2019 study using TRAIL-resistant mouse breast cancer cells (2.5 mg/kg TRAIL per injection) [36]. Future studies should investigate dosing with different concentrations of TRAIL liposomes to establish a dose response and maximum tolerated dose in mice. If necessary, EST liposomes can be combined with known TRAIL sensitizers such as taxanes [57, 58] curcumin [59], piperlongamine [60], or Yoda1 [61, 62].

Although NOD.SCID mouse models are acceptable for representing prostate cancer progression, the lack of a fully functioning immune system minimizes important cross-talk between innate and adaptive immune cells. Additionally, macrophages in this model may also be hypofunctional and other antigen presenting cells not fully mature or differentiated which may have affected their localization of the chosen markers. Future studies should explore leukocyte infiltration using a syngeneic prostate cancer mouse model, such as engrafting B6CaP cells in C57BL/6 mice [63]. Alternatively, and perhaps most translationally relevant, these human prostate tumors can be inoculated in mice with humanized immune systems [64]. While our study provides new understanding of the spatial distribution of the predominant innate inflammatory cell types in these types of xenografts, inclusion of adaptive immune cells will allow for a greater understanding of T cell, B cell, and NK cell infiltration and treatment interactions within these cell types.

## Conclusion

Prostate tumors contain multiple infiltrating leukocytes that subdue or aid tumor progression in response to local inflammation. There have been extensive studies examining the immune profile of prostatic tumors. However, no study has investigated immune cell spatial organization with nanoscale cargo in a NOD.SCID orthotopic prostate cancer model. For the first time, we demonstrated here that tumor-associated macrophages are the most abundant immune cell group in orthotopic prostate xenografts with a spatial distribution that evolves according to tumor growth time, size, and EST liposome treatment. We were able to identify TAMs coated with EST liposomes in solid orthotopic prostate xenografts, demonstrating that these liposomes are capable of infiltrating and inducing apoptosis within the tumor microenvironment. This work provides an important proof-of-concept for harnessing innate immune cells for the liposomal delivery of TRAIL to prostate tumors. Furthermore, macrophage infiltration seen in these orthotopic prostate xenografts provides a groundwork for understanding immune landscapes within this NOD.SCID model and beyond.

## Data Availability

The raw data supporting the conclusion of this article will be made available by the authors, without undue reservation.
