# Supplementary material for: Spatial distribution of tumor-associated macrophages in an orthotopic prostate cancer mouse model
Source: Pathol Oncol Res. 2024 Apr 16;30:1611586. doi: 10.3389/pore.2024.1611586 (PMC11058651; doi:10.3389/pore.2024.1611586)
Supplement: Supplementary file 1 [file DataSheet3.docx]

**QuPath Tumor Margins Script**

/**

* Script to help with annotating tumor regions, separating the tumor margin from the center.

*

* Here, each of the margin regions is approximately 500 microns in width.

*

* @author Pete Bankhead

*/

import qupath.lib.common.GeneralTools

import qupath.lib.objects.PathAnnotationObject

import qupath.lib.objects.PathObject

import qupath.lib.roi.PathROIToolsAwt

import java.awt.Rectangle

import java.awt.geom.Area

import static qupath.lib.scripting.QPEx.*

//-----

// Some things you might want to change

// How much to expand each region

double expandMarginMicrons = 500.0

// Define the colors

def coloInnerMargin = getColorRGB(0, 0, 200)

def colorOuterMargin = getColorRGB(0, 200, 0)

def colorCentral = getColorRGB(0, 0, 0)

// Choose whether to lock the annotations or not (it's generally a good idea to avoid accidentally moving them)

def lockAnnotations = true

//-----

// Extract the main info we need

def imageData = getCurrentImageData()

def hierarchy = imageData.getHierarchy()

def server = imageData.getServer()

// We need the pixel size

if (!server.hasPixelSizeMicrons()) {

print 'We need the pixel size information here!'

return

}

if (!GeneralTools.almostTheSame(server.getPixelWidthMicrons(), server.getPixelHeightMicrons(), 0.0001)) {

print 'Warning! The pixel width & height are different; the average of both will be used'

}

// Get annotation & detections

def annotations = getAnnotationObjects()

def selected = getSelectedObject()

if (selected == null || !selected.isAnnotation()) {

print 'Please select an annotation object!'

return

}

// We need one selected annotation as a starting point; if we have other annotations, they will constrain the output

annotations.remove(selected)

// If we have at most one other annotation, it represents the tissue

Area areaTissue

PathObject tissueAnnotation

if (annotations.isEmpty()) {

areaTissue = new Area(new Rectangle(0, 0, server.getWidth(), server.getHeight()))

} else if (annotations.size() == 1) {

tissueAnnotation = annotations.get(0)

areaTissue = PathROIToolsAwt.getArea(tissueAnnotation.getROI())

} else {

print 'Sorry, this script only support one selected annotation for the tumor region, and at most one other annotation to constrain the expansion'

return

}

// Calculate how much to expand

double expandPixels = expandMarginMicrons / server.getAveragedPixelSizeMicrons()

def roiOriginal = selected.getROI()

def areaTumor = PathROIToolsAwt.getArea(roiOriginal)

// Get the outer margin area

def areaOuter = PathROIToolsAwt.shapeMorphology(areaTumor, expandPixels)

areaOuter.subtract(areaTumor)

areaOuter.intersect(areaTissue)

def roiOuter = PathROIToolsAwt.getShapeROI(areaOuter, roiOriginal.getC(), roiOriginal.getZ(), roiOriginal.getT())

def annotationOuter = new PathAnnotationObject(roiOuter)

annotationOuter.setName("Outer margin")

annotationOuter.setColorRGB(colorOuterMargin)

// Get the central area

def areaCentral = PathROIToolsAwt.shapeMorphology(areaTumor, -expandPixels)

areaCentral.intersect(areaTissue)

def roiCentral = PathROIToolsAwt.getShapeROI(areaCentral, roiOriginal.getC(), roiOriginal.getZ(), roiOriginal.getT())

def annotationCentral = new PathAnnotationObject(roiCentral)

annotationCentral.setName("Center")

annotationCentral.setColorRGB(colorCentral)

// Get the inner margin area

areaInner = areaTumor

areaInner.subtract(areaCentral)

areaInner.intersect(areaTissue)

def roiInner = PathROIToolsAwt.getShapeROI(areaInner, roiOriginal.getC(), roiOriginal.getZ(), roiOriginal.getT())

def annotationInner = new PathAnnotationObject(roiInner)

annotationInner.setName("Inner margin")

annotationInner.setColorRGB(coloInnerMargin)

// Add the annotations

hierarchy.getSelectionModel().clearSelection()

hierarchy.removeObject(selected, true)

def annotationsToAdd = [annotationOuter, annotationInner, annotationCentral];

annotationsToAdd.each {it.setLocked(lockAnnotations)}

if (tissueAnnotation == null) {

hierarchy.addPathObjects(annotationsToAdd, false)

} else {

tissueAnnotation.addPathObjects(annotationsToAdd)

hierarchy.fireHierarchyChangedEvent(this, tissueAnnotation)

if (lockAnnotations)

tissueAnnotation.setLocked(true)

}

**Supplementary Figure 6**: QuPath tumor margins script.
