# Supplementary material for: Spatial distribution of tumor-associated macrophages in an orthotopic prostate cancer mouse model
Source: Pathol Oncol Res. 2024 Apr 16;30:1611586. doi: 10.3389/pore.2024.1611586 (PMC11058651; doi:10.3389/pore.2024.1611586)
Supplement: Supplementary file 2 [file DataSheet2.docx]

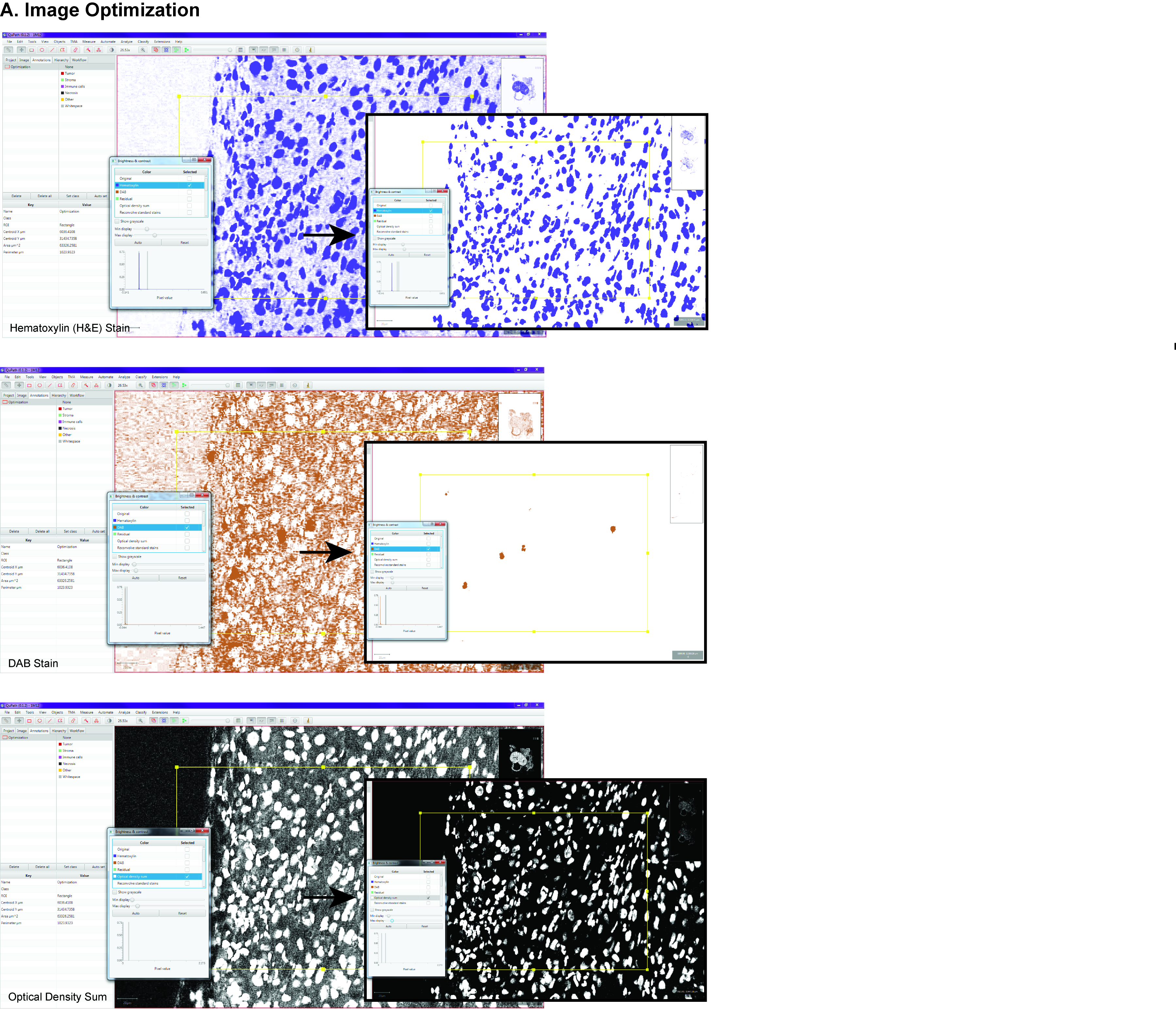


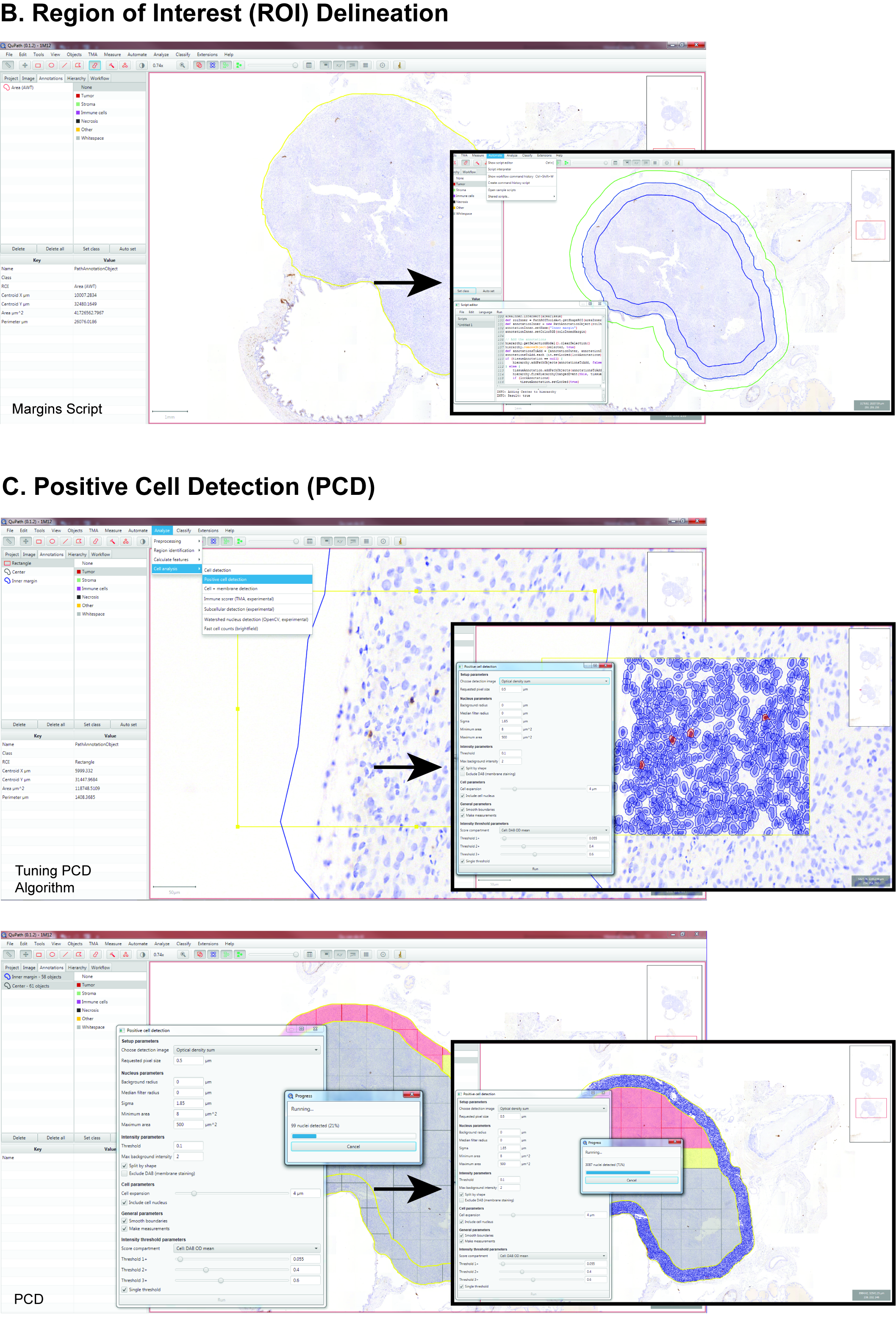

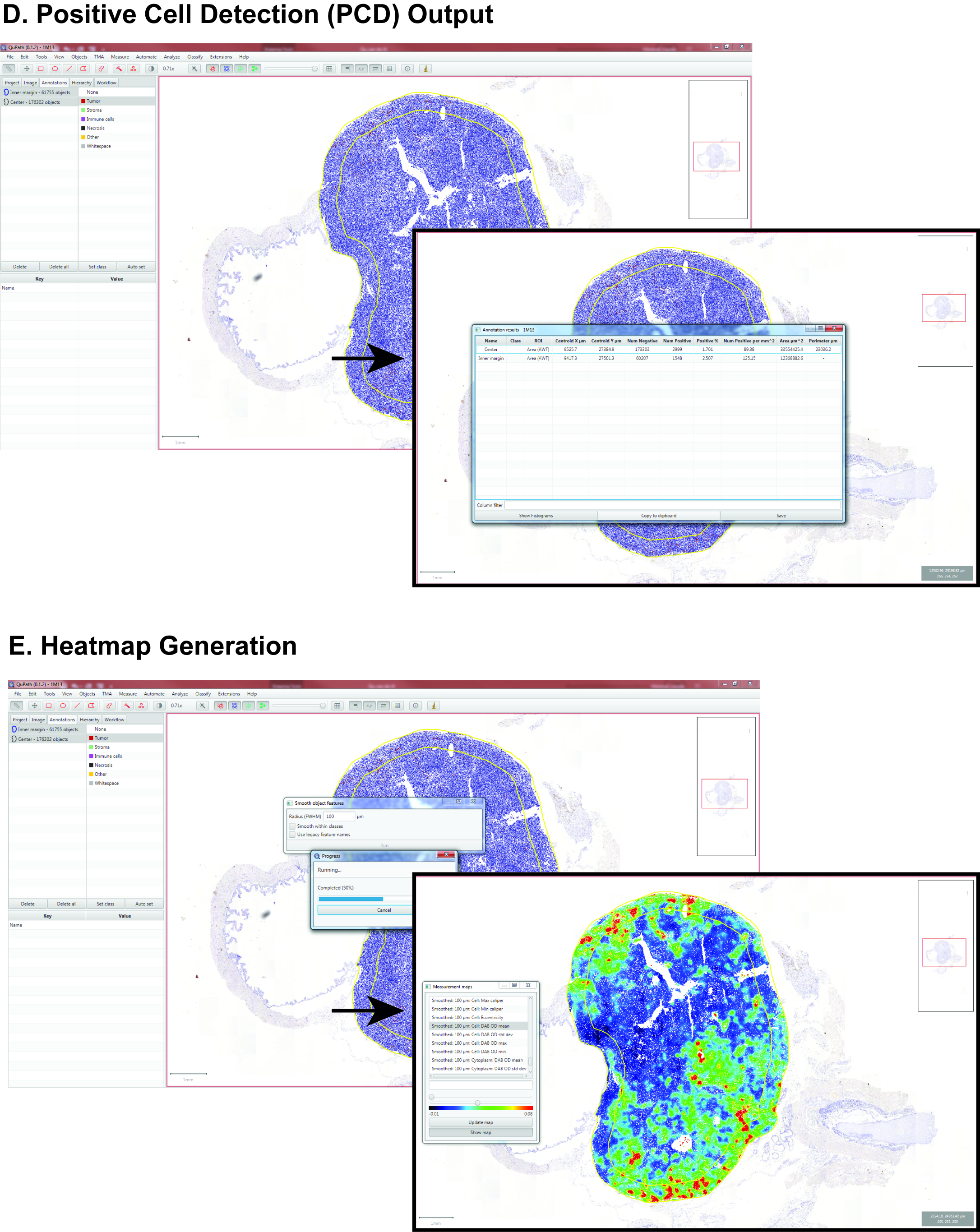


**Supplementary Figure 5**: QuPath project example on mouse prostate tumor section. **A.** Stain separation and optimization using color deconvolution to digitally separate Hematoxylin-eosin (H&E) stain (cell nuclei, extracellular matrix, and cytoplasm), 3,3’-diamonobenzidine (DAB) stain (nucleic acids and proteins), and residual from RGB image. **B.** Manual region of interest (ROI) annotation and margin script execution to delineate inner and marginal tumor areas. **C.** Positive cell detection (PCD) algorithm use for inner and marginal tumor areas. **D.** PCD results table with measurements of all cells. **E.** Density heatmap generation.
