# Supplementary material for: Spatial distribution of tumor-associated macrophages in an orthotopic prostate cancer mouse model
Source: Pathol Oncol Res. 2024 Apr 16;30:1611586. doi: 10.3389/pore.2024.1611586 (PMC11058651; doi:10.3389/pore.2024.1611586)
Supplement: Supplementary file 3 [file DataSheet1.docx]

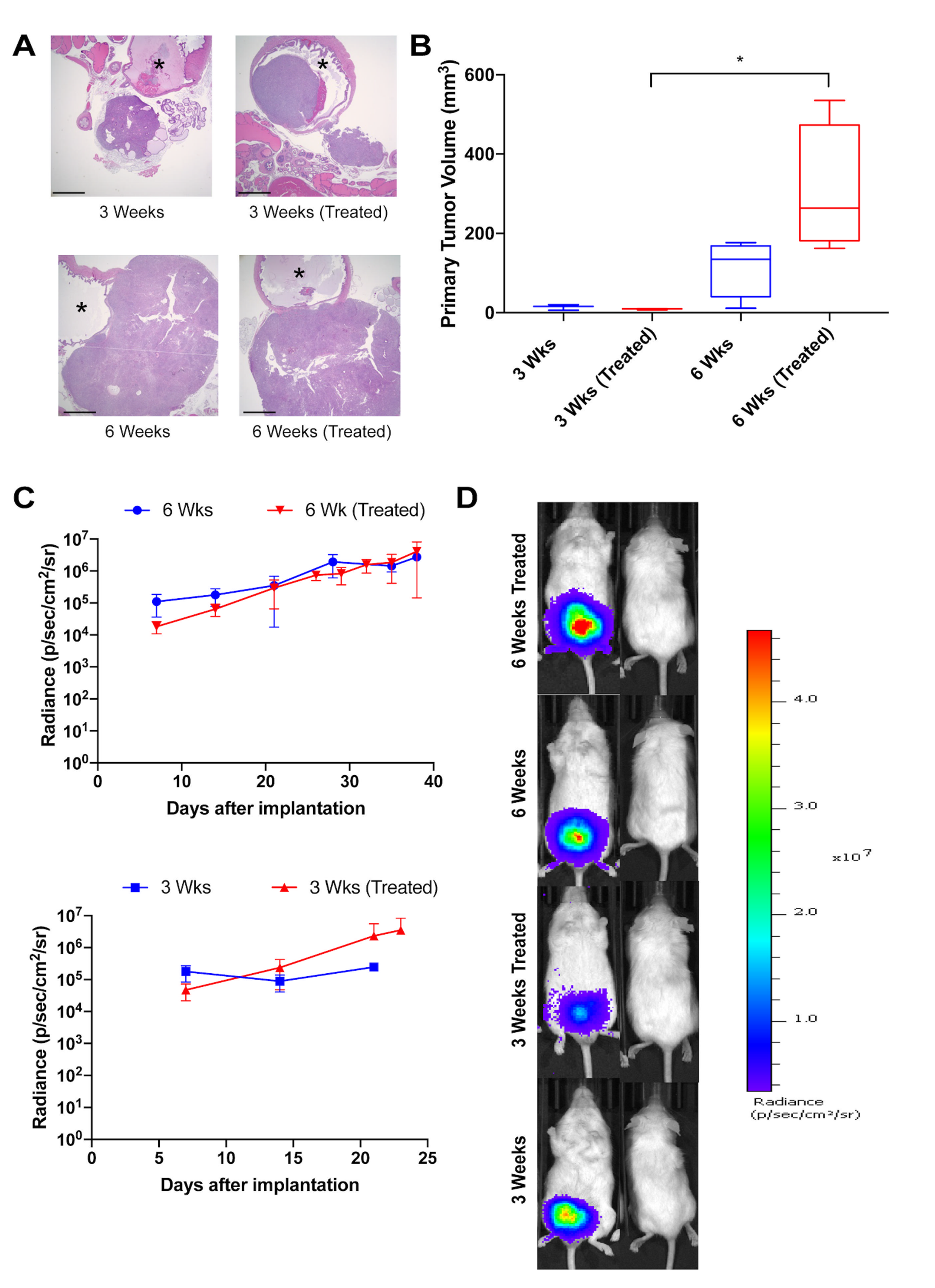


**Supplementary Figure 1**: Orthotopic prostate xenograft measurements. **A.** Representative H&E staining of a xenograft from each treatment cohort. Asterisk (*) indicates urinary bladder lumen for orientation. Scale bar = 50 μm. **B.** Xenograft tumor volumes. **C.** Average radiance from xenografts of 3 weeks vs 3 weeks (treated) and 6 weeks vs 6 weeks (treated). **D.** Whole animal BLI of ventral (left) and dorsal (right) side of representative mice from each treatment group. The values represent the mean ± SD (n= 3 or 4). *p < 0.05.


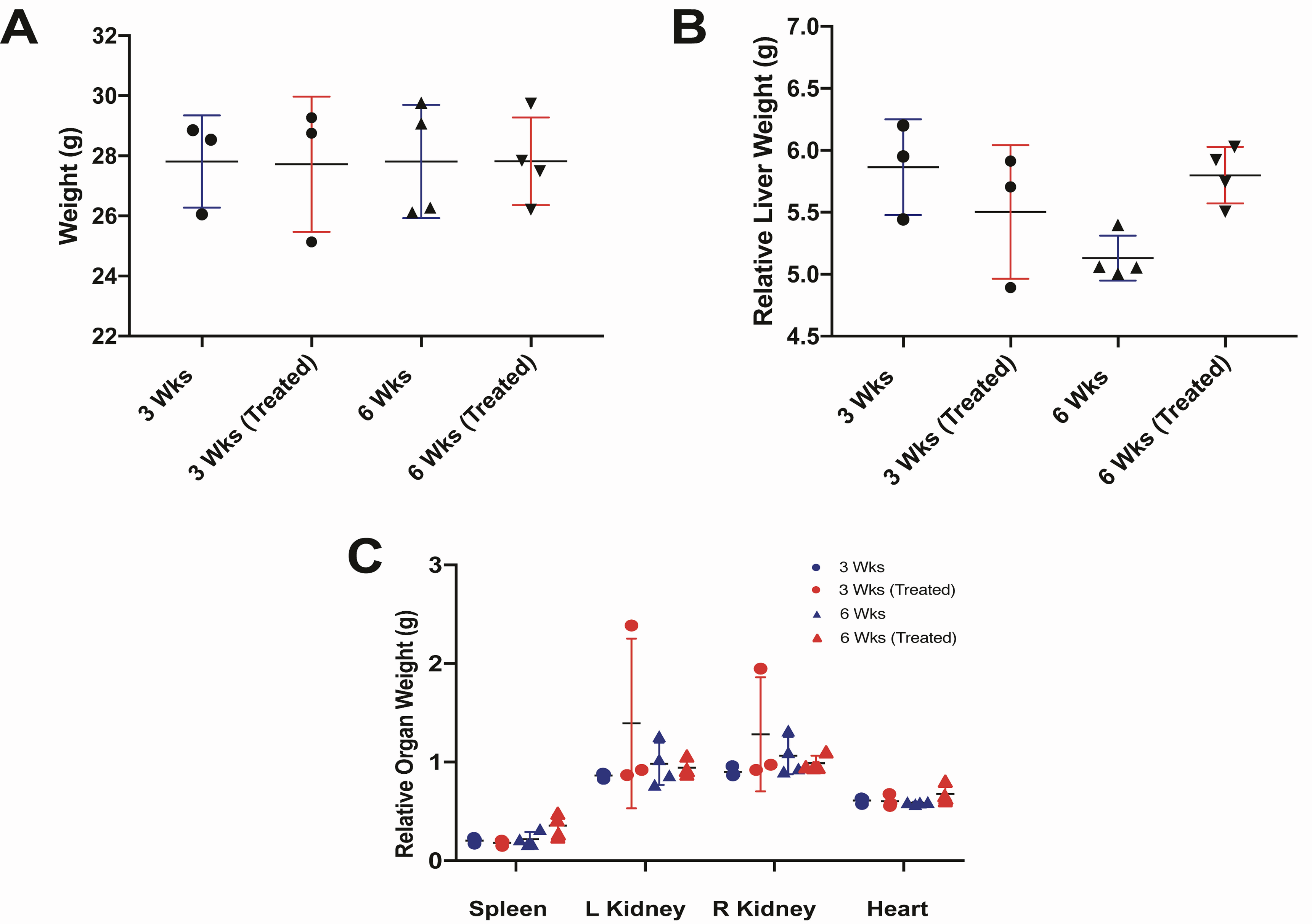


**Supplementary Figure 2**: Terminal body and organ weights. **A.** Body weights of animals at the end of study. **B.** Weight of excised liver relative to body weight. **C.** Relative organ weights of mice in each group post-sac. The values represent the mean ± SD (n= 3 or 4).


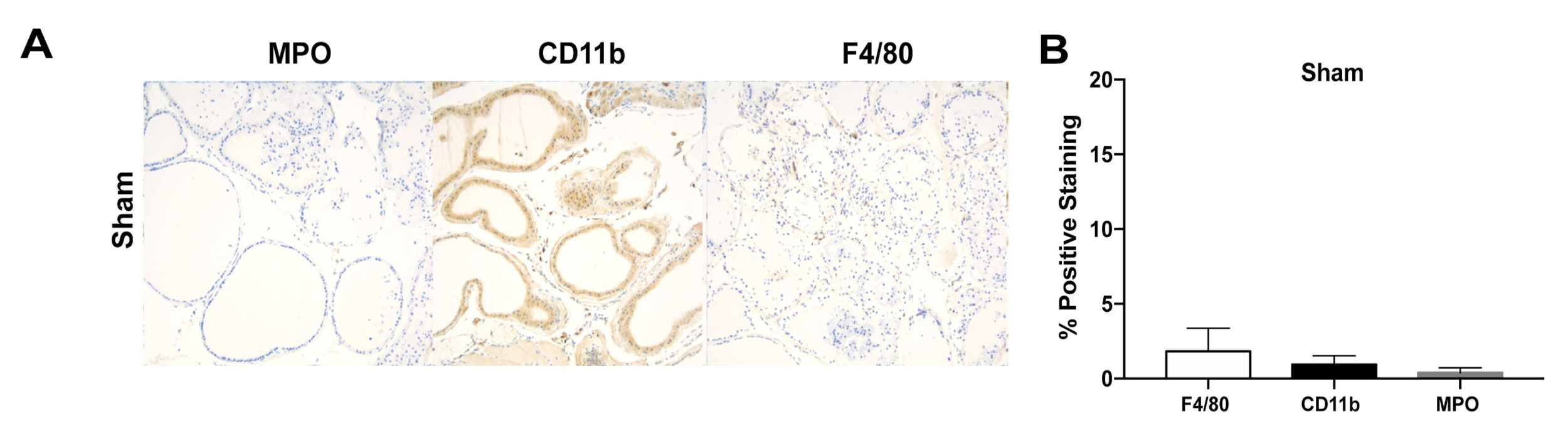


**Supplementary Figure 3**: IHC staining in sham (control) group for different leukocyte populations. **A.** IHC staining of mouse ventral prostate tissue subjected to sham surgery. MPO = myeloperoxidase/neutrophil granulocyte marker, CD11b = myeloid marker, F4/80 = macrophage marker. **B.** Quantification of percent positive staining (n=3).


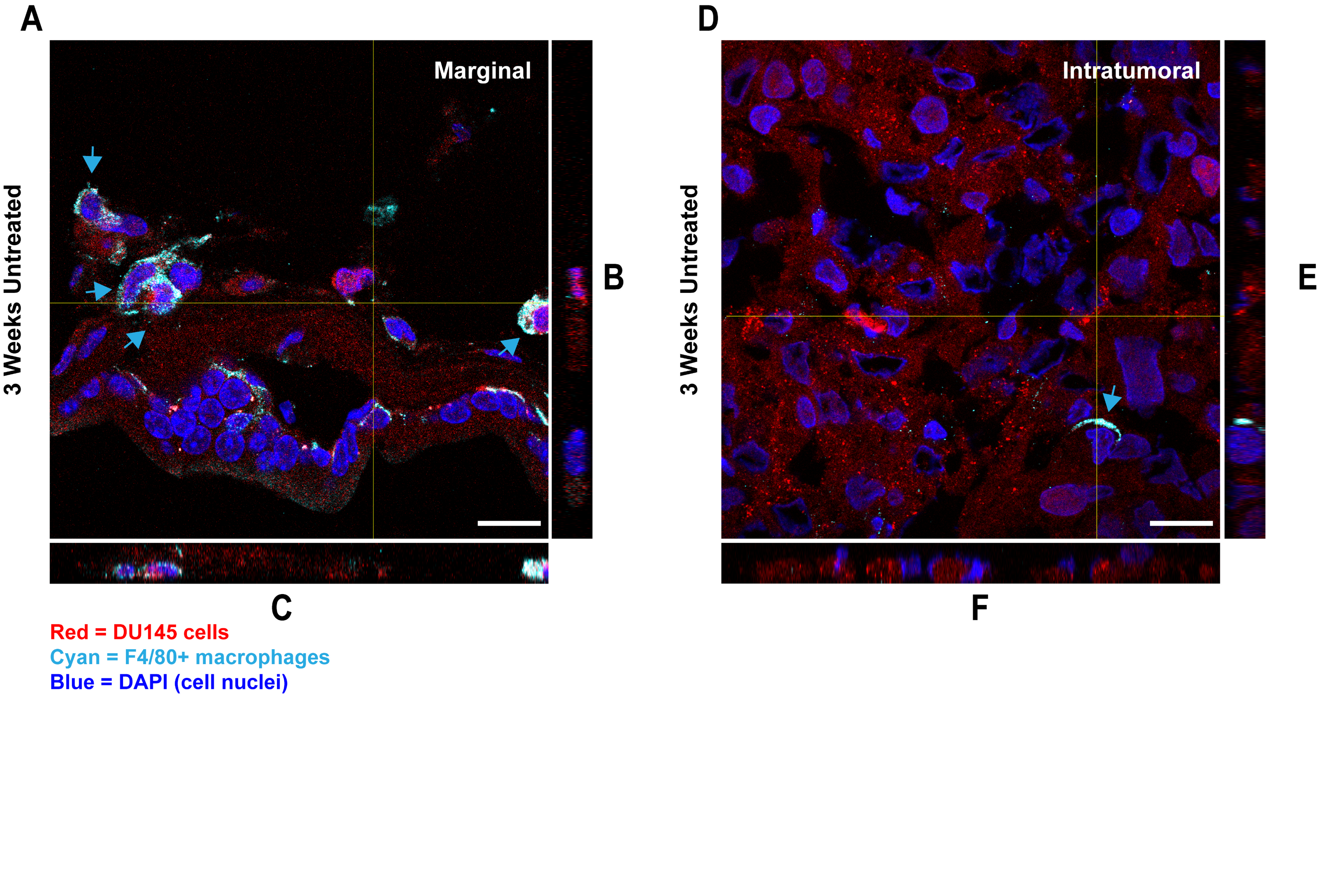


**Supplementary Figure 4**: Negative control images depicting macrophage infiltration alone. Cancer cells are labeled with mCherry (red), macrophages are labeled with Alexa Fluor^®^ 647 Conjugate (cyan), cell nuclei are labeled with DAPI (blue), EST liposomes are labeled with TopFluor (green). Blue arrows represent F4/80+ macrophages. **A.** Z-stack composite photomicrograph of marginal TAMS. **B.** Z-projection in Y-Z direction. **C.** S-projection in X-Z direction. **D.** Z-stack composite photomicrograph of intratumoral TAMS. **E.** Z-projection in Y-Z direction. **F.** S-projection in X-Z direction. Scale bar = 20 μm.

| **Primary antibody** | | | | | **Secondary antibody** | | |
| --- | --- | --- | --- | --- | --- | --- | --- |
| **Name** | **Antigen retrieval and blocking** | **Manufacturer** | **Concentration** | **Incubation time** | **Name and manufacturer** | **Concentration** | **Incubation time** |
| F4/80 (NB600-404) | Proteinase K (Dako) - 5 min | Novus Biologicals LLC, Littleton, CO | 1:900 | 1 hr | Rabbit anti-rat (BA-4001, Vector Laboratories, Inc.) | 1:2000 | 15 min |
| CD11b (NB110-89474) | Epitope Retrieval 2 solution (Leica) - 20 min; Protein Block (Ref# x0909, Dako) - 10 min | Novus Biologicals LLC | 1:15,000 | 1 hr | Bond Polymer Refine Detection system (Leica) | Ready-to-use | n/a |
| MPO (A0398) | Epitope Retrieval 2 solution (Leica) - 20 min; Protein Block (Ref# x0909, Dako) - 10 min | Dako, Carpinteria, CA | 1:4000 | 1 hr | Bond Polymer Refine Detection system (Leica) | Ready-to-use | n/a |
| MBP | Proteinase K (Dako) - 5 min | Lee Laboratory, Mayo Clinic | 1:1000 | 1 hr | Rabbit anti-rat secondary (BA-4001, Vector Laboratories, Inc.) | 1:2000 | 15 min |
| Neutro (ab2557) | Epitope Retrieval 2 solution (Leica) - 20 min | Abcam, Cambridge, MA | 1:2000 | 1 hr | Rabbit anti-rat secondary (BA-4000, Vector Laboratories, Inc.) | 1:2000 | 15 min |

**Supplementary Table 1**: Chromogenic immunohistochemistry antibodies.
